# Rosmarinic acid treatment during porcine oocyte maturation attenuates oxidative stress and improves subsequent embryo development *in vitro*

**DOI:** 10.7717/peerj.6930

**Published:** 2019-06-18

**Authors:** Yan Zhang, Jing Guo, Xiao Wei Nie, Zi Yue Li, Yu Meng Wang, Shuang Liang, Suo Li

**Affiliations:** 1 College of Animal Science and Technology, Jilin Agricultural University, Changchun, China; 2 Department of Animal Science, Chungbuk National University, Cheongju, South Korea; 3 Chongqing Reproductive and Genetics Institute, Chongqing, China; 4 Chongqing Key Laboratory of Human Embryo Engineering, Chongqing, China; 5 Department of Reproductive Medicine, Affiliated Hospital of Nanjing University of Chinese Medicine, Nanjing, China; 6 Department of Animal Science, College of Animal Sciences, Jilin University, Changchun, China

**Keywords:** Rosmarinic acid, Porcine oocyte, *In vitro* maturation, Oxidative stress

## Abstract

**Background:**

*In vitro* maturation (IVM) of oocytes has been widely used in the field of assisted reproductive technology. However, oocytes can be injured by oxidative stress during the process of IVM.

**Methods:**

The present study was designed to evaluate the influences of rosmarinic acid (RA) on the IVM of porcine oocytes and the subsequent development of early-stage embryos as well as its underlying mechanisms. Various concentrations of RA (5 µM, 10 µM, and 25 µM) were treated with porcine oocyte maturation medium during the period of IVM.

**Results and Discussion:**

The results showed that 5 µM RA treatment during the period of porcine oocyte IVM improves blastocyst quality and hatching ability after parthenogenetic activation. Furthermore, the presence of RA during the period of IVM dramatically improved the total number of cells after somatic cell nuclear transfer compared to the number of cells in the control group. Notably, RA treatment during the period of porcine oocyte IVM decreased intracellular reactive oxygen species generation not only in oocytes but also in cumulus cells. Further analysis showed that the intracellular free thiols levels in the oocytes were enhanced by treatment with RA during the period of porcine oocyte IVM compared to the free thiols levels in the control groups. These results indicate that RA improves the developmental competence of porcine oocytes during the IVM period by attenuating oxidative stress.

## Introduction

*In vitro* maturation (IVM) of oocytes refers to the process in which immature oocytes obtained from ovaries are cultured under the appropriate conditions *in vitro* so that they undergo a series of complex physiological and biochemical transformations, ultimately developing into mature oocytes that are able to be fertilized (Lonergan & Fair, 2016; Schultz & Wassarman, 1977). IVM of oocytes is a pivotal step in assisted reproductive technology (ART) and has broad applications in helping infertility patients and improving the breeding and reproductive efficiency of livestock (Chian et al., 2009; Gil et al., 2010; Hoelker et al., 2017; Jin et al., 2014). However, the quality and developmental competence of *in vitro* matured oocytes remains low compared with that of *in vivo* matured oocytes (Rizos et al., 2002; Sutton, Gilchrist & Thompson, 2003). The main reason may be that the unstable extracellular environments during IVM, resulting in the maturation of the nucleus and cytoplasm, are not synchronized (Funahashi & Day, 1993; Zhao et al., 2014). The oocytes continuously produce reactive oxygen species (ROS) during growth and development, and the IVM process is likely to produce additional ROS due to mechanical handling, air, light and other factors (Cetica et al., 2001; Kitagawa et al., 2004; Morado et al., 2013; Waiz et al., 2016). Although ROS are a normal product of cell metabolism, excessive ROS generation will cause DNA damage, mitochondrial dysfunction, lipid peroxidation and protein oxidation modification, thus deteriorating oocytes and blocking their subsequent developmental potential (Rajani et al., 2012; Tamura et al., 2008). Antioxidants can bind to oxygen free radicals, thus inhibiting the initiation of free radical chain propagation, eliminating the damage caused by ROS (Agarwal & Majzoub, 2017; Kitagawa et al., 2004). Under oocyte *in vitro* culture conditions, various types of antioxidants have been added as supplements either alone or in different combinations to attenuate oxidative stress, improving oocyte quality (Liang et al., 2018; Wu et al., 2011; Xu et al., 2018; Yu et al., 2018). Pigs are very important livestock animals for biomedical research and agricultural production. However, porcine oocytes are more sensitive to oxidative stress than those of other livestock animals such as cattle and sheep (Somfai et al., 2011). Therefore, reducing harmful ROS is an effective means of improving the quality of *in vitro* matured porcine oocytes and increasing their developmental potential.

Rosmarinic acid (RA) is a water-soluble polyphenol and a naturally occurring hydroxylated compound commonly found in various medicinal herbs of the family Lamiaceae (Pereira et al., 2005; Zhang et al., 2014). A growing body of evidence shows multiple beneficial effects of RA on different animal cells, including antioxidant, antimutagenic, anti-inflammatory and radical-scavenging properties (Huang & Zheng, 2006; Kantar Gok et al., 2018; Lee et al., 2008; Makino et al., 2002; McKay & Blumberg, 2006). Previous studies have identified RA as one of the most potential antioxidants in the hydroxycinnamic acid group of polyphenols (Ghaffari et al., 2014; Soobrattee et al., 2005). It has been indicated that supplementation with RA not only increases glutathione levels but also activates antioxidant enzyme activity in *in vitro* cell studies (Chkhikvishvili et al., 2013; Fallarini et al., 2009; Kantar Gok et al., 2018). Recent research has shown that RA can improve boar sperm quality during cryopreservation by protecting against oxidative stress (Luno et al., 2014).

Although the beneficial biological functions of RA are well documented, the effect of RA during oocyte maturation has not been investigated to date. Here, we hypothesized that RA treatment of porcine oocytes during the IVM period would improve the quality of the oocytes and improve their subsequent *in vitro* developmental potential. In the present study, we first investigated the effect of RA on porcine oocyte nuclear maturation and subsequent embryonic development competence after parthenogenetic activation (PA) and somatic cell nuclear transfer (SCNT). Subsequently, the mechanism underlying the effect of RA on porcine oocyte maturation was determined empirically.

## Materials and Methods

All chemicals used in this study were purchased from Sigma-Aldrich (St Louis, MO, USA) unless noted otherwise.

### Collection and IVM of porcine oocytes

Ovaries were obtained from slaughtered pre-pubertal gilts at a local slaughterhouse and transported to the laboratory in sterile saline in a vacuum flask. Cumulus-oocyte complexes (COCs) were aspirated from 3–6 mm antral follicles, and those showing a multilayer of compact cumulus cells and homogeneous ooplasm were specifically collected using Tyrode’s lactate–hydroxyethylpiperazine ethane sulfonic acid (HEPES) medium with 0.1% polyvinyl alcohol (PVA, w/v) and 0.05 g/L gentamycin under a stereomicroscope. The COCs were cultured in tissue culture medium 199 (TCM-199, Invitrogen, Carlsbad, CA, USA) supplemented with 10% (v/v) porcine follicular fluid, 0.91 mM Na pyruvate, 1% penicillin G-streptomycin sulfate, 0.5 µg/mL follicle stimulating hormone, and 0.5 µg/mL luteinizing hormone. The maturation medium was covered with mineral oil and incubated at 38.5 °C in an atmosphere containing 5% CO_2_ at 100% humidity for 42 h. The RA stock solution was diluted with TCM-199 (2.5 mM) and stored in the dark at −20 °C.

### PA, SCNT and *in vitro* culture

PA and SCNT were performed according to our previously described procedures (Liang et al., 2018; Liang et al., 2017b). Porcine ear fibroblasts were isolated and used as nuclear donors. After PA and SCNT, the activated oocytes or reconstructed embryos were cultured in bicarbonate-buffered PZM-5 containing 4 mg/mL BSA and 7.5 µg/mL cytochalasin B for 3 h to suppress extrusion of the pseudo-second polar body. Next, the activated oocytes or reconstructed embryos were thoroughly washed and cultured in bicarbonate-buffered PZM-5 supplemented with 4 mg/mL BSA for 7 days at 38.5 °C in an atmosphere containing 5% CO_2_ at 100% humidity. The cleavage rate was examined on day 2 after activation. Blastocyst diameter, formation and hatching rates were examined on day 7 after activation. The total number of cells in blastocysts was determined by staining the cells with 10 µg/mL Hoechst 33342 for 15 min and counting the cells that exhibited blue fluorescence under a fluorescence microscope (IX70, Olympus, Tokyo, Japan).

### Measurement of intracellular ROS and free thiols levels in oocytes

Oocyte intracellular ROS and free thiols levels were measured by incubating the cells with 10 µM 2′,7′-dichlorodihydrofluorescein diacetate (Invitrogen, NY, USA) and 10 µM 4-chloromethyl-6,8-difluoro-7-hydroxycoumarin (Invitrogen) for 15 min and 30 min, respectively. The fluorescence signal was captured as TIFF files using a digital camera (DP72; Olympus) connected to a fluorescence microscope (IX70, Olympus). NIH ImageJ software (National Institutes of Health, Bethesda, MD, USA) was used to analyse the fluorescence intensities of the oocytes.

### Measurement of intracellular ROS levels in cumulus cells via flow cytometry

At the end of IVM, cumulus cells were removed from the oocyte by treatment with 1 mg/mL hyaluronidase and collected in a 1.5-ml centrifugal tube. At least 50 COCs were collected from each group. The intracellular ROS content of the cumulus cells was measured with 10 µM 2′,7′-dichlorodihydrofluorescein diacetate (Invitrogen, NY, USA) and analysed by flow cytometry.

### Statistical analyses

Statistical analyses were performed using analysis of variance (ANOVA), and data obtained from two groups were compared using the Student’s *t*-test embedded in GraphPad Prism version 6.01(GraphPad Software, La Jolla, CA, USA). The number of independent replicates (Re) of each experiment is shown in the figure legends. The data were expressed as the mean ± standard error of the mean (SEM). A *p* value of <0.05 was considered significant.

## Results

### Effects of RA treatment during IVM on porcine oocyte maturation and subsequent *in vitro* embryo development after PA

**Figure 1:**
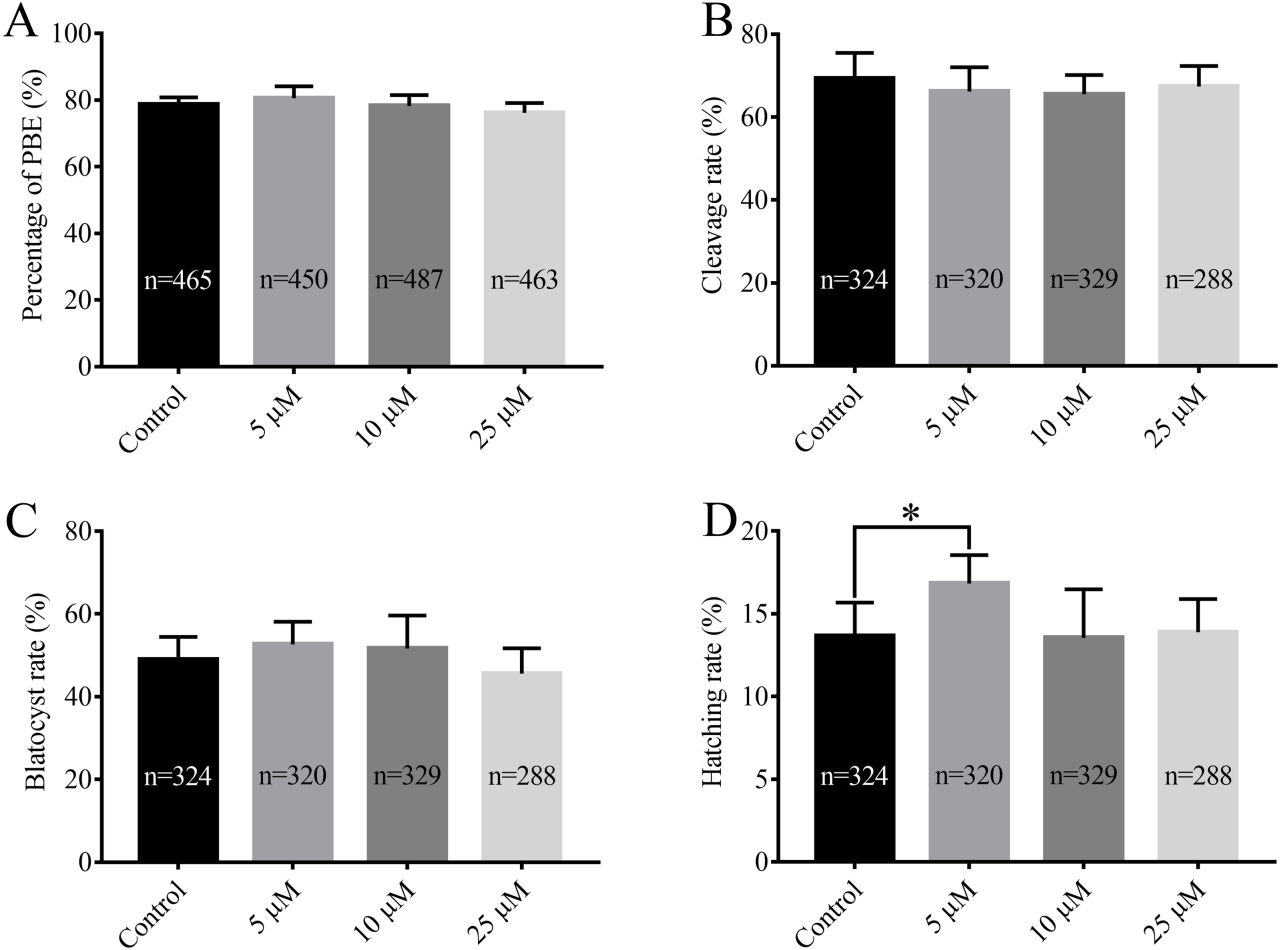
Effects of RA at various concentrations during IVM on porcine oocyte PBE and subsequent *in vitro* development of parthenogenetic embryos. (A) PBE rate. Re = 8. (B) Cleavage rate, (C) blastocyst rate and (D) hatching rate after parthenogenetic activation. Re = 7. *p < 0.05.

**Figure 2:**
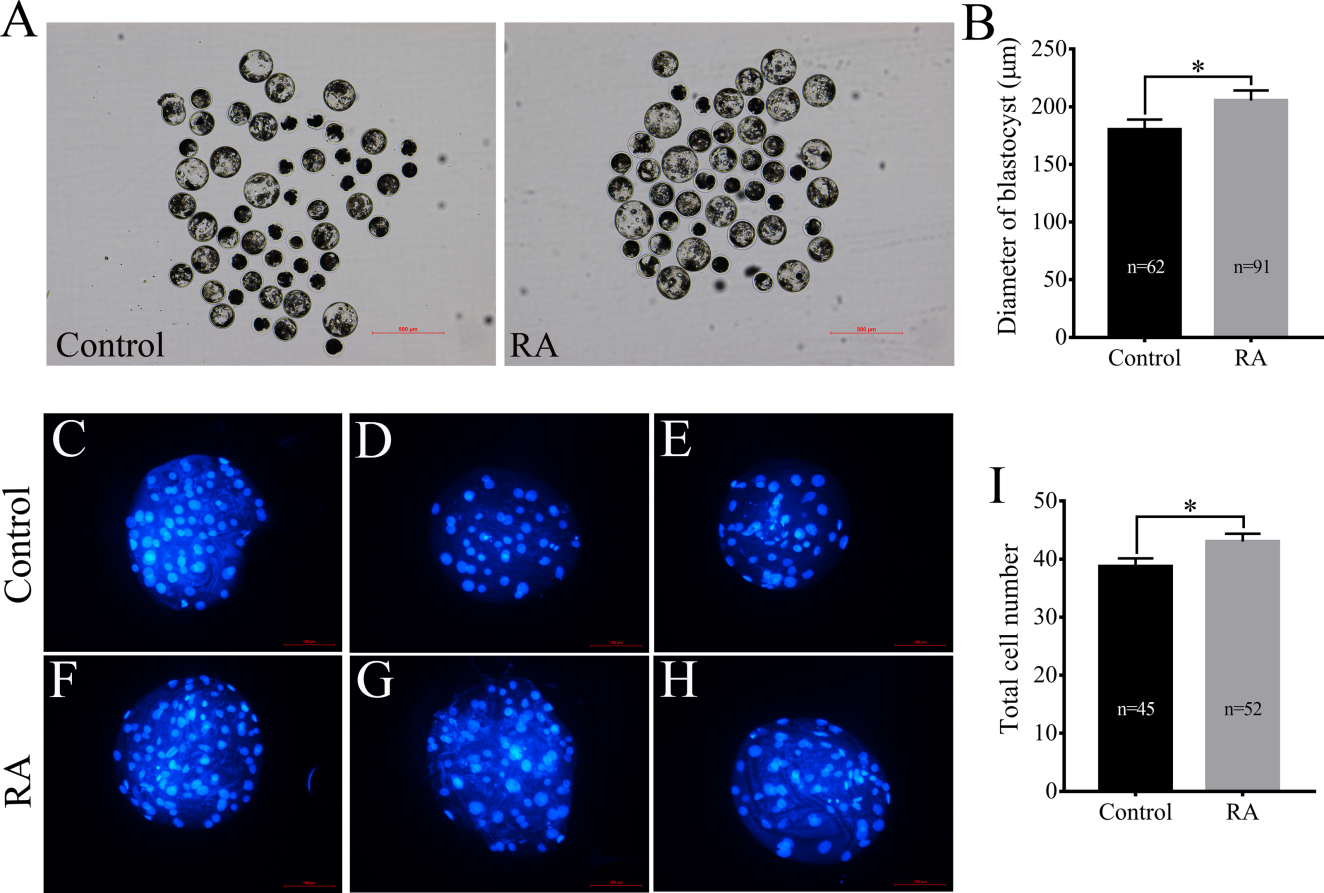
Effects of RA treatment during IVM on the of quality blastocysts. (A) and (B) Average diameter of blastocysts on day 6 in the control and RA treatment groups. Scale bar = 500 µm; Re = 4. (C-H) and (I) Average total cell count of blastocysts on day 7 in the control and RA treatment groups. Scale bar = 100 µm; Re = 3. *p < 0.05.

After being collected, immature porcine oocytes were cultured with various concentrations of RA. The polar body extrusion (PBE) rates were examined after 42 h of IVM. Figure 1A shows that PBE rates were not affected by treatment with 5 µM, 10 µM, or 25 µM RA (78.69 ± 2.13%, 80.54 ± 3.61%, 78.22  ± 3.24%, and 76.18 ± 2.95%, respectively). After PA, the ability of embryos to develop *in vitro* was analysed. As shown in Figs. 1B and 1C, we did not observe significant differences in the cleavage rate (69.34 ± 6.20%, 66.20 ± 5.86%, 66.53 ± 4.61%, and 67.40 ± 5.00%, respectively) or blastocyst formation rate (48.94 ± 5.23%, 52.64 ± 5.46%, 51.67 ± 7.95%, and 45.61 ± 6.09%, respectively) between the control and RA-treated groups during the IVM period. We further investigated the ability of the blastocysts to hatch; the results are shown in Fig. 1D. The results revealed that treatment with 5 µM RA during the IVM period significantly increased the rate of blastocyst hatching compared to that of the control group (16.83 ± 1.72% *vs.* 13.67 ± 2.01%; *p* < 0.05). Further analysis showed that treatment with 5 µM RA during the IVM period sharply increased the diameter of blastocysts (205.4 ± 4.39 vs. 180.4 ± 4.29; *p* < 0.05; Figs. 2A and 2B) and the total number of cells in blastocysts (43.04 ± 1.34 *vs.* 38.77 ± 1.37; *p* < 0.05; Figs. 2C–2I).

### Effects of RA treatment during IVM on *in vitro* development of SCNT embryos

**Figure 3:**
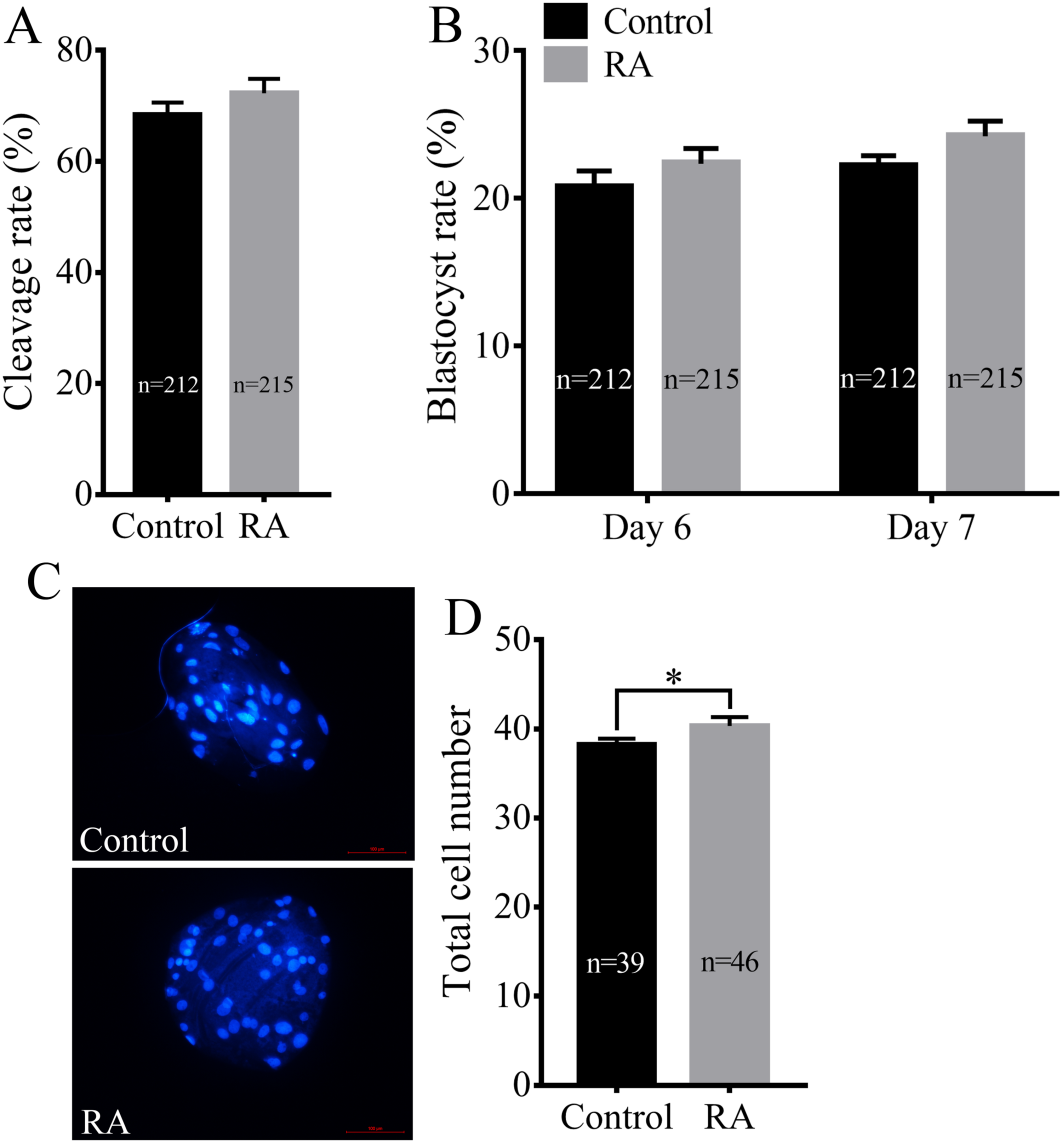
Effects of RA treatment during IVM on the developmental competence of porcine SCNT embryos. (A) Cleavage rate of SCNT embryos in the control and RA treatment groups. (B) Blastocyst formation rate in the control and RA treatment groups on days 6 and 7. (C) and (D) Average total cell count of blastocysts on day 7 in the control and RA treatment groups. Re = 3. *p < 0.05.

We next assayed whether RA treatment during the IVM period improved the developmental competence of porcine SCNT embryos. Consistent with the above results, RA treatment during the IVM period did not significantly increase the cleavage rate of SCNT embryos (72.30 ± 2.59% *vs.* 68.38 ± 2.22%; Fig. 3A). We further examined the blastocyst formation rate of SCNT embryos on days 6 and 7. Although the blastocyst formation rate was increased on day 6 (22.35 ± 1.02% *vs.* 20.74 ± 1.11%) or day 7 (24.20 ± 1.03% *vs*. 22.16 ± 0.73%), the results showed that there was no significant difference between the RA treatment and control groups (Fig. 3B). Compared with the control group, the treated group showed obvious effects of RA on SCNT quality: RA treatment during the IVM period remarkably increased the total number of cells in blastocysts (40.33 ± 1.00 *vs*. 38.26 ± 0.66; *p* <0.05; Figs. 3C and 3D).

### Effects of RA treatment during the IVM period on intracellular ROS levels in porcine oocytes and cumulus cells

**Figure 4:**
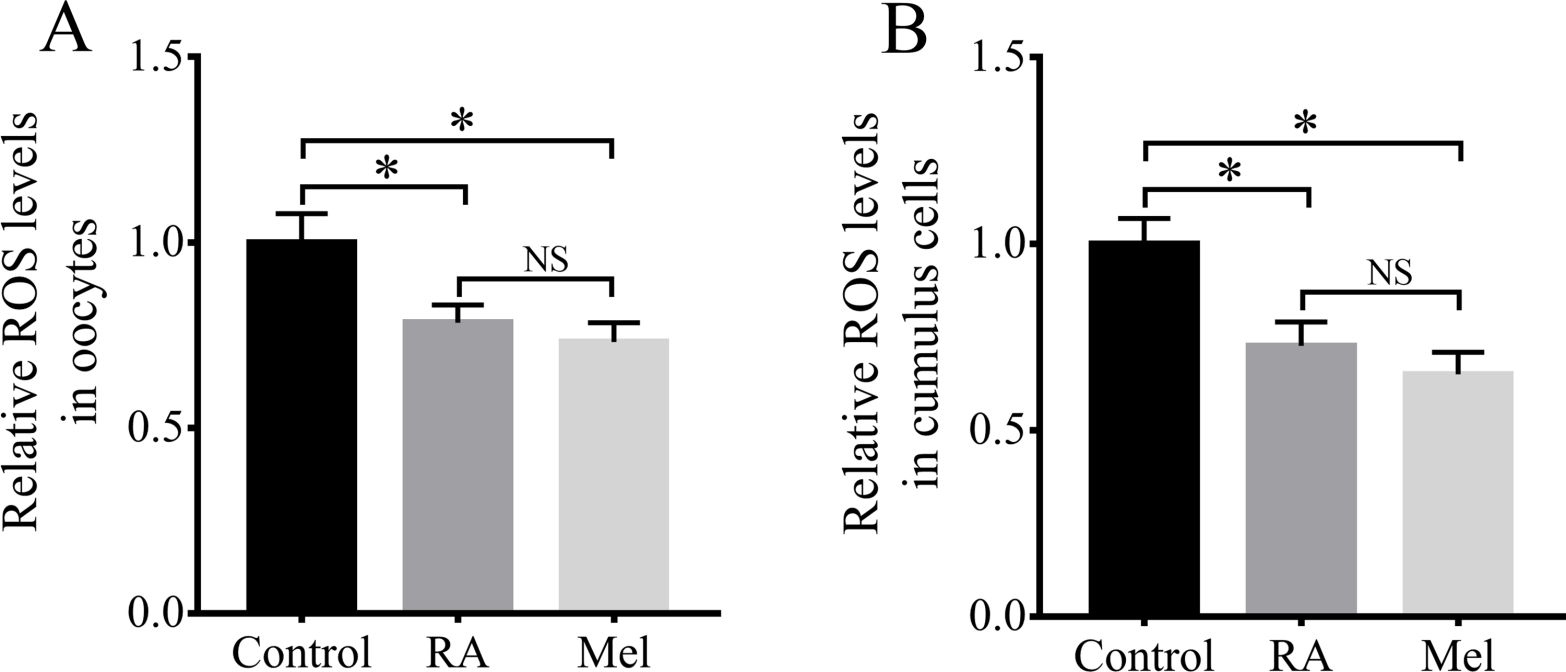
RA treatment during IVM attenuated intracellular ROS levels in porcine oocytes and cumulus cells. Relative intracellular ROS levels in oocytes (A; Re = 3) and cumulus cells (B; Re = 4). Mel: Melatonin. *p < 0.05.

Because RA has antioxidant properties, we examined whether RA treatment during the IVM period would improve the resistance of porcine oocytes to oxidative stress. The results revealed that there were significantly lower intracellular ROS levels in the oocytes (Fig. 4A) and cumulus cells (Fig. 4B) of the RA treatment group than in those of the control group that was not treated with RA during the IVM period. When compared with the control group, the levels of ROS in the oocytes were much lower in the melatonin (Mel, 1 µM) and RA treatment group, whereas it showed no significant changes between RA and Mel treatment groups. In addition, to further determine the effect of RA against oxidative stress, the oocytes were pre-incubated with RA for 3 h. After that, H_2_O_2_ (50 µM) was added for 30 min. As shown in Fig. S1, the H_2_O_2_ and RA + H_2_O_2_ treatment group showed a higher intracellular ROS levels compared to the control group. However, the levels of intracellular ROS levels was no significant changes in the oocytes between RA and RA + H_2_O_2_ treatment group.

### Effects of RA treatment during the IVM period on intracellular free thiols levels in porcine oocytes

**Figure 5:**
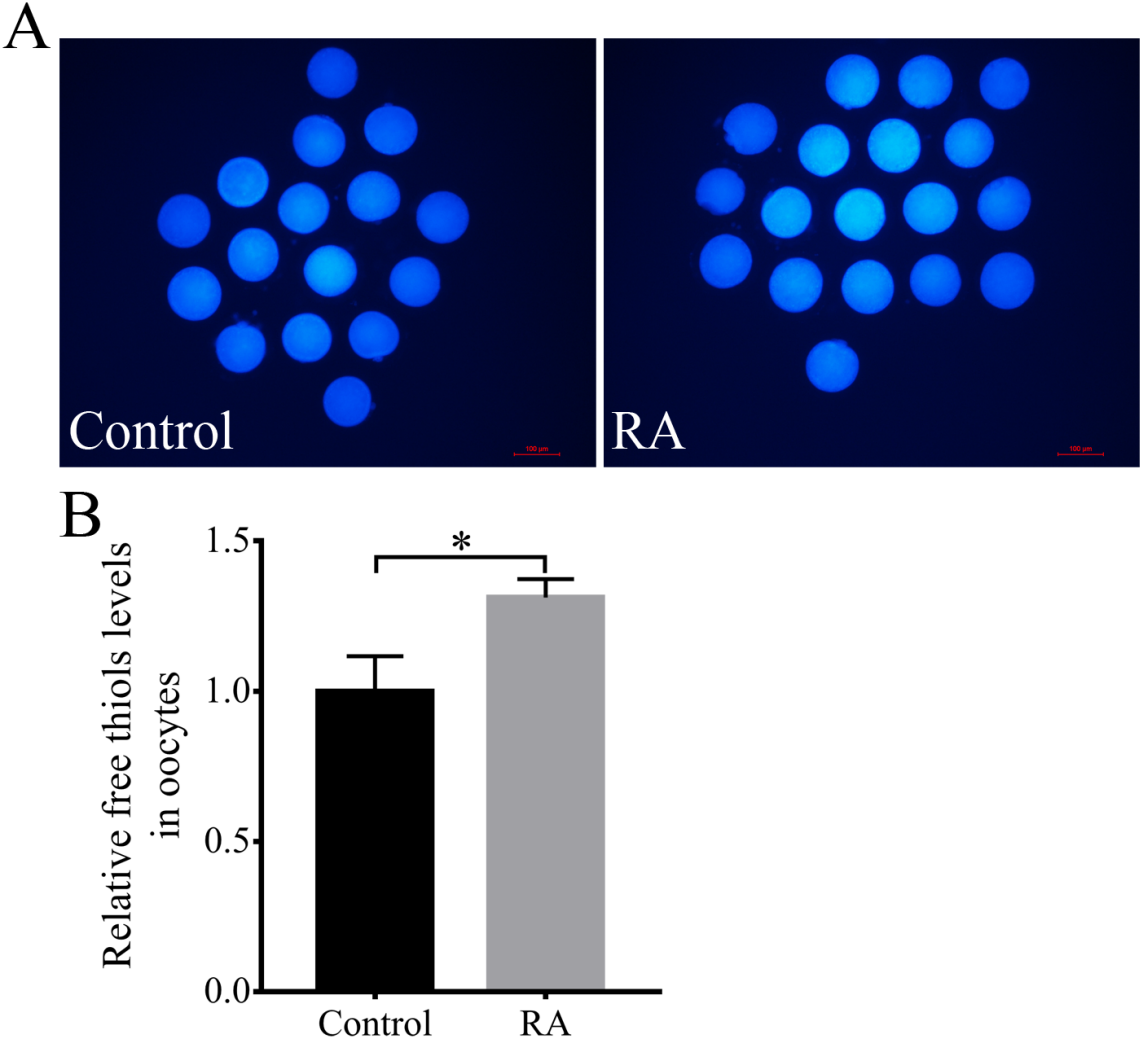
RA treatment during IVM improves intracellular free thiols levels in porcine oocytes. (A) Representative fluorescent images of intracellular free thiols levels in oocytes from the control and RA treatment groups. Scale bar = 100 µm. (B) Relative intracellular free thiols levels in oocytes. Re = 3. *p < 0.05.

Next, the intracellular free thiols levels in the porcine oocytes were evaluated at the end of the IVM period. As shown in Figs. 5A and 5B, the intracellular free thiols levels were obviously increased in the RA treatment group compared to the control group without RA treatment during the IVM period.

## Discussion

IVM of oocytes is closely associated with the enhancement of oxidative stress production, which induces various impairments in oocytes and causes developmental competence to decline (Liang et al., 2017a; Tamura et al., 2008). In the present study, the effects of RA supplementation on IVM of porcine oocytes were evaluated. The results of the current study indicate that RA supplementation during IVM of porcine oocytes has beneficial effects on the quality of the oocytes, leading to improved embryo developmental competence after PA and SCNT.

IVM of oocytes consists of artificially simulating the *in vivo* environment in which the immature oocytes grow and develop into mature ones and discharge the first polar body (Holzer et al., 2007; Sun & Nagai, 2003). Oocyte IVM is a highly significant stage of *in vitro* embryo production. When the quality of an *in vitro* matured oocyte is improved, its developmental competence is also greatly influenced. In the current study, we found that 5 µM RA treatment during the process of IVM markedly improved the quality of porcine PA and SCNT embryos, as indicated by increased blastocyst hatching and total cell counts. Unexpectedly, however, RA treatment during the process of IVM did not affect PBE by porcine oocytes. Further analyses revealed that RA treatment during the process of IVM also did not affect the cleavage rate or the blastocyst formation rate after PA or SCNT. These results partly support our hypothesis, which is that RA treatment during the process of IVM improves the *in vitro* developmental potential of porcine oocytes. In the future, more data will be obtained from *in vitro* fertilization and embryo transfer to detect the developmental potential of RA treated oocytes.

Compared to *in vivo* matured oocytes, *in vitro* matured oocytes are faced with increased oxidative pressure and are susceptible to oxidative stress (Combelles, Gupta & Agarwal, 2009). Under normal physiological conditions, the ROS and antioxidants in the body are in a state of dynamic balance (Valko et al., 2007). If there are more ROS than antioxidants, oxidative stress will result. During the process of IVM, COCs are at an elevated risk of being stimulated by exogenous factors that break the dynamic balance of redox homeostasis in oocytes (Han et al., 2006; Hu et al., 2001). This imbalance of homeostasis severely restricts the ability of oocytes to mature *in vitro* and affects their subsequent embryo developmental competency (Ali, Bilodeau & Sirard, 2003; Prasad et al., 2016). Porcine oocytes contain a large amount of cytoplasmic lipids, which are particularly sensitive to ROS (Dunning, Russell & Robker, 2014; McKeegan & Sturmey, 2011). Thus, antioxidant supplementation could control cascades of uncontrolled oxidation and protect oocytes from oxidative damage by scavenging ROS during IVM (Ali, Bilodeau & Sirard, 2003; Liang et al., 2018). RA, as powerful natural antioxidant, has powerful ROS scavenging activities and is an auxiliary factor in several important antioxidant enzyme systems (Rong, Liang & Niu, 2018). The antioxidant mechanism of RA is related to its chemical structure, in which the carboxylic acid group and the catechol structure in the aromatic ring work together to neutralize free radicals (Benavente-García et al., 1997; Luno et al., 2014). A previous study showed that RA could suppress H_2_O_2_-induced cytotoxicity in N2A cells by reducing intracellular ROS levels (Ghaffari et al., 2014). Moreover, RA also effectively attenuated amyloid- *β*-induced lipid hydroperoxide and ROS accumulation in PC12 cells (Iuvone et al., 2006). Luno et al. (2014) have shown that RA can effectively promote the function and fertilizing ability of sperm in cattle by preventing lipid peroxidation and DNA oxidation. A recent study has shown that RA supplementation improves sperm DNA integrity during a freeze-drying procedure (Olaciregui et al., 2017). Consistently, in the present study, RA treatment during the process of IVM effectively reduced intracellular ROS levels in porcine oocytes and cumulus cells at the end of IVM. This result is consistent with our hypothesis that RA can improve porcine oocyte quality by mitigating impairments caused by oxidative stress.

To further evaluate the underlying process and mechanism through which RA improves the quality and development potential of porcine oocytes, we examined intracellular free thiols levels. One of the markers of cytoplasmic maturation of oocytes at the end of IVM is intracellular free thiols levels (De Matos & Furnus, 2000; Funahashi et al., 1994). Several studies have shown that oocytes with higher intracellular ROS levels have lower intracellular free thiols levels, and their subsequent embryonic development potential is also insufficient (Liang et al., 2018; Liang, Nie & Zhao, 2017c; Wu et al., 2011). Previous studies have suggested that RA, which is one of the most effective antioxidants in the hydroxycinnamic acid group of polyphenols (Soobrattee et al., 2005), can protect Jurkat T cells and human neuroblastoma cells against oxidative stress and cause intracellular free thiols levels to increase (Chkhikvishvili et al., 2013; Fallarini et al., 2009). In the present study, RA treatment during the process of IVM promoted the accumulation of free thiols in the cytoplasm. This finding further supports our hypothesis that RA treatment during the process of IVM improves the quality and development potential of porcine oocytes after PA and SCNT.

## Conclusion

The present study demonstrates that RA during the process of IVM protects porcine oocytes against oxidative stress and improves their subsequent *in vitro* developmental competence. Therefore, RA is a potential candidate antioxidant agent for oocytes during the process of IVM and will be helpful for improving the efficiency of *in vitro* embryo production in pigs.

##  Supplemental Information

10.7717/peerj.6930/supp-1 Figure S1 Intracellular ROS levels in the oocytes after H_2_O_2_ exposure Relative intracellular ROS levels in oocytes. Re = 3. *p < 0.05; **p < 0.01.

10.7717/peerj.6930/supp-2 Data S1 Raw data for the statistic analysis Raw data applied for data analyses and preparation for Figure 1A, 1B, 1C and 1D; Figure 2B and 2I; Figure 3A, 3B and 3D; Figure 4A and 4B; Figure 5B and Figure S1.

## Additional Information and Declarations

### Data Availability

The following information was supplied regarding data availability: The raw data is available in the Supplemental File.
