# Supplementary figures and images for: Rosmarinic acid treatment during porcine oocyte maturation attenuates oxidative stress and improves subsequent embryo development in vitro
Source: PeerJ. 2019 Jun 18;7:e6930. doi: 10.7717/peerj.6930 (PMC6587974; doi:10.7717/peerj.6930)

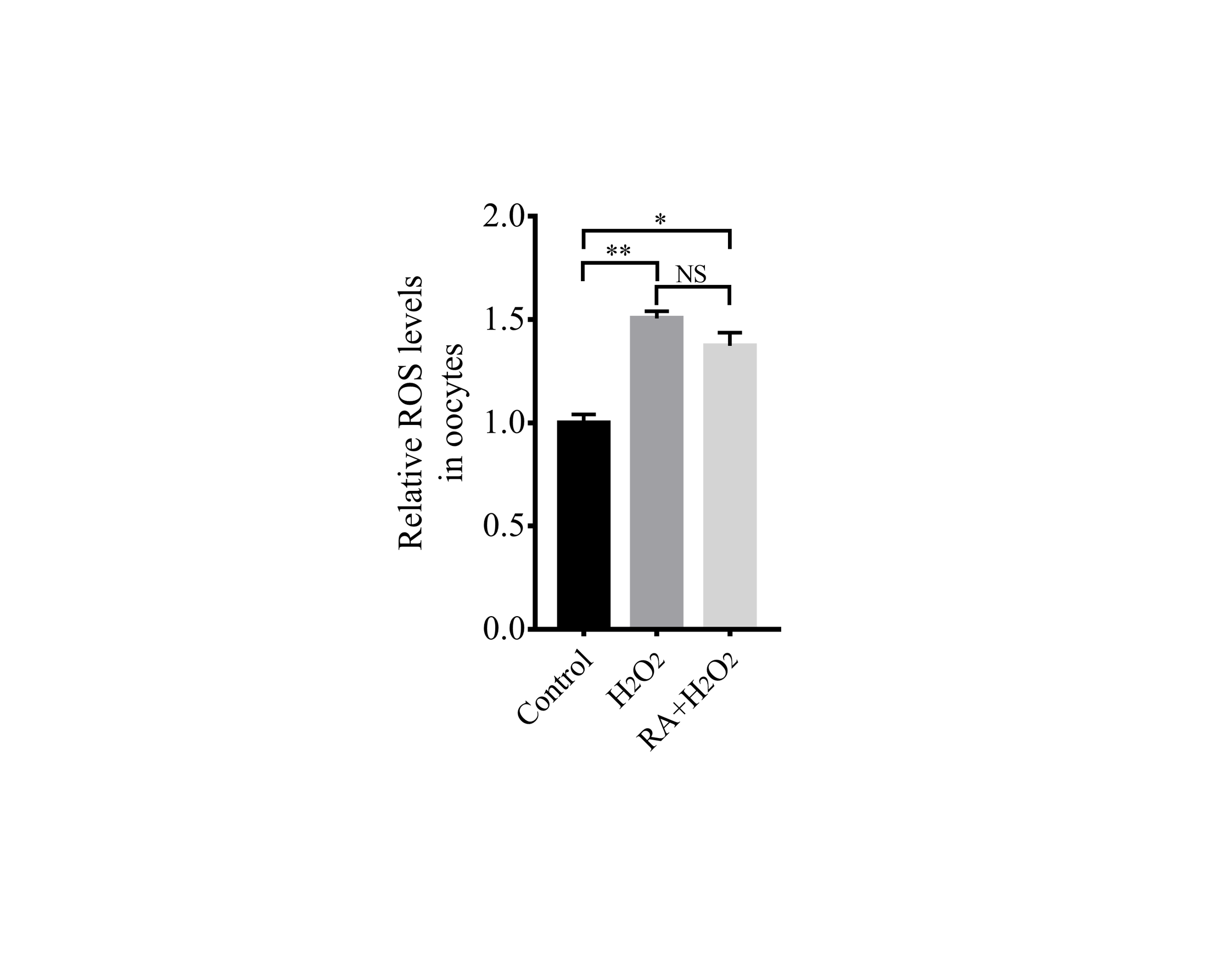

Supplement: Figure S1 — Relative intracellular ROS levels in oocytes. Re = 3. *p < 0.05; **p < 0.01. [file peerj-07-6930-s001.png]
